# A Social Media Single Session Intervention Designed to Promote Healthier Social Media Use: Youth Focus Group Feedback

**DOI:** 10.2196/73780

**Published:** 2026-03-02

**Authors:** Jane Harness, Sarah E Domoff, Heide Rollings, Jessica L Schleider

**Affiliations:** 1 Department of Psychiatry, University of Michigan–Ann Arbor, 1500 East Medical Center, Ann Arbor, MI, United States, 1 (734) 764-0231; 2 Department of Psychology, University at Albany, State University of New York, Albany, NY, United States; 3 Michigan State University, East Lansing, MI, United States; 4 Pine Rest Christian Mental Health Services, Grand Rapids, MI, United States; 5 Department of Medical Social Sciences, Northwestern University Feinberg School of Medicine, Chicago, IL, United States

**Keywords:** social media, youth, mental health, single session intervention, online, web

## Abstract

**Background:**

The effects of social media use on an individual’s well- or ill-being are highly contextualized to the individual’s characteristics and their specific type of social media use. Youth have variable levels of insight about how social media affects them, knowledge about different ways they can change their use, and self-efficacy to make changes. Interventions designed to promote youth modifications of their social media use are needed to minimize harm in an individualized manner.

**Objective:**

The objective of this study was to first develop a Social Media Single Session Intervention (SM SSI) intended to improve insight about effects of social media use on well/ill-being, knowledge of potential changes to make and self-efficacy to make changes and to gather youth feedback related to the SSI’s acceptability, feasibility, perceived helpfulness and how it could best be implemented.

**Methods:**

A total of 3 (one in-person and two virtual) focus groups were held between June and October 2024. Feedback from 7 youth aged 16‐20 (average age 17.43, SD 1.62) years was collected and summarized.

**Results:**

Summarized feedback from the focus groups indicated that the SM SSI is acceptable, feasible, and has the potential to help young people. Youth gave recommendations to include information about additional social media applications, to include an additional peer story exploring other specific challenges, and to include crisis resources. They generally enjoyed going through it together as a group and thought people would use it if assigned by school or a health provider or if they could earn credit for it.

**Conclusions:**

Requested changes will be incorporated into a final iteration of the SM SSI to be piloted in a future study. This initial feedback shows promise that youth would find value in, learn from, and change behavior as a result of this SSI.

## Introduction

Research demonstrating both increases in ill-being and well-being associated with social media use led to conceptualizing social media influence on two separate spectra [1]. Because social media experiences and users are unique, efforts have shifted towards education and intervention [2]. Some youth are aware of how social media affects them and have made changes to their use due to this awareness [3]; however, some youth may lack this insight, lack knowledge of potential changes to make, or lack the self-efficacy to make those changes. Because digital single session interventions (SSIs) are both highly accessible and effective for youth mental health concerns [4-6], we designed a SSI to improve youth insight about how social media affects them, knowledge about potential changes to make, and self-efficacy to make those changes. Key components of digital SSI’s include relevant science summary, establishing user authority in the topic, user summarization of knowledge gained, and stories from peers [7]. Digital SSIs are uniquely feasible as they remove barriers such as privacy concerns, transportation, and time. Motivational interviewing (MI) is a therapeutic gold standard approach to encourage behavior change [8,9] and has strengths in adaptability to digital platforms and autonomy promotion [10]. Therefore, we took an MI-informed approach to create a SSI which invites ambivalence by exploring user likes and dislikes about social media use. This self-guided intervention complements school- [11] and clinician-based [12] educational interventions. The primary objective of this study was to gather youth feedback related to the SSI’s acceptability. We were also interested in youth feedback about the SSI’s feasibility, perceived helpfulness, and how it could best be implemented.

## Methods

### Procedure

Focus group participants (n=7) from a youth advisory board were hosted in person and virtually between June and Oct 2024. Focus groups lasted for 1‐2 hours and were recorded. The in-person focus group was hosted at the youth advisory board’s usual meeting place and the virtual focus groups were hosted on a secure institutional Zoom meeting. One adult leader of the youth advisory board was present for all of the focus groups in addition to the PI and consultants. Inclusion criteria were age 14‐24 years and membership of a youth advisory board. After consent was obtained, youth were asked a few questions and then the digital social media single session intervention (SM SSI) was projected as feedback was requested page by page. Youth were asked questions at the end of the intervention to gather additional feedback. Please refer to Multimedia Appendix 1 for the full list of pre- and post-SSI questions.

### Intervention

**Figure 1.**
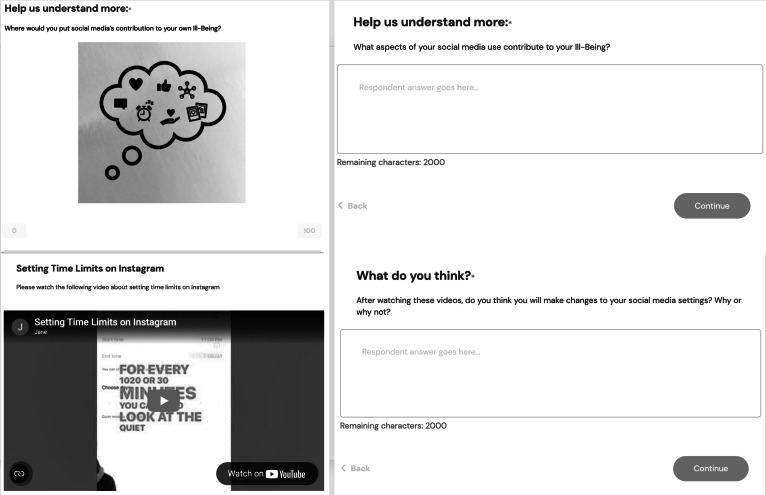
Example Images of the SM SSI. SM SSI: Social Media Single Session Intervention.

The SM SSI was created on Computerized Intervention Authoring System (CIAS)[16]. Figure 1 demonstrates screenshots of the SM SSI. The intervention is a self-guided 30-minute virtual experience. The intervention introduces thinking about social media’s influence on both well-being and ill-being on separate spectrums with examples. Next, users are invited to read two vignettes about a teen’s specific social media experience, quantify and qualify the social media experience’s contribution to the teen’s well/ill-being and devise advice for the teen in the story. Next, users are invited to think about social media’s contribution to their own well/ill-being, positive and negative aspects and what they like and dislike about social media. They are asked what (if anything) they have done anything to change their social media use in the past and why. They are invited to watch 3 short (30‐61 s) videos about how to turn off like and view counts, how to set time limits, and how to change the content they are presented with on Instagram. They are asked if they think they will make changes to their social media settings and why or why not. Development of the SSI was guided by examples from Dr. Schleider’s lab as she was a consultant for this project.

### Participants

Participants were recruited from a youth advisory board. The advisory board leaders distributed study information and consent and assent forms to group members via email. Members were encouraged to read all the information and ask any questions via email to the study and advisory board leaders. Interested individuals let the advisory board leader know that they would like to participate and arrangements were made for either in person (one total on 6/26/2024) or virtual (two total on 10/22/2024 and 10/27/2024) focus groups.

### Ethical Considerations

The University of Michigan Institutional Review Board approved this study (HUM00242288). Parental consent was not necessary due to the minimal risk of the study. Guardians were allowed access to the SSI if they wished, but there were no requests. We reviewed the IRB approved consent form and electronic consent was obtained from all minor participants at the beginning of each focus group. Participants were compensated $100. Measures taken to promote privacy and confidentiality included separate collection of identifying information necessary for compensation purposes and removing identifying information from the transcripts.

### Data Analysis

Because of our small sample size, we did not use formal qualitative analysis of the transcripts. Transcripts from each focus group were combined and organized in chronological order by question and page of the SSI by the PI (JH). Feedback was further collapsed into key themes using an inductive approach. Topics were discussed among the other authors who attended the focus groups virtually (SED and HR).

## Results

### Demographic Information

A total of 7 youth provided feedback about the SM SSI. The average age of focus group participant was 17.43 (SD 1.62; range 16‐20) years. Our sample was 42.8% Asian (3/7), 28.6% Multiracial (Asian/White) (2/7), 14.2% Black (1/7), and 14.2% White (1/7); 100% female and 100% not Hispanic or Latinx. Feedback was organized into the three following categories.

### Acceptability of the SM SSI

Youth participants reported that they viewed the SM SSI and its content as helpful and enjoyable. They felt that the stories were realistic, although names should be changed. Participants shared that they would recommend it to all their friends or specific friends. They found the videos to be useful and even learned new information, with participants sharing that the how-to videos encourage change. Focus group participants appreciated that the tone of the SSI was partnership around shared goals rather than it being overly commanding.

### Recommended Changes to the SM SSI

Participants wanted one more story exploring comparison with peers on social media and concerns about their “digital footprint.” They wanted additional videos related to TikTok, Snapchat and artificial intelligence. One participant recommended including 988 and crisis information along with a link tree of other resources such as “OK2SAY” and additional videos. They also recommended adding information about the science related to social media and youth mental health. Feedback on smaller details included a less computer-like narrator, smoother transitions between the sections, deleting the captions in the videos (as they could be turned on if needed) and removing the video narrator from the screen.

### Implementation Recommendations

Some participants said that they might leave the free response questions blank. This led to discussions about how they preferred to go through it in a small group like we did during the focus group, with one participant sharing it was comforting to know they were not alone in thinking about this topic. Participants did not think that people would click on a social media advertisement for the SSI due to the prevalence of false advertising on social media, but would engage if it was assigned by their school. One person thought that it would be a good precollege class registration requirement. A QR code for the SSI on a poster at school or work would be feasible. They suggested that people could earn a certificate to be displayed on their LinkedIn page if it was offered there. One participant suggested SM SSI administration in a clinical setting by a doctor or therapist.

## Discussion

The focus group feedback gathered during this study shows promise that youth would find value in, learn from, and change behavior as a result of this SSI. They appreciated the collaborative style over a commanding or didactic style. The main recommendations were to make further additions. The stylistic recommendations will be incorporated into the next iteration of this SSI to be put forward for further piloting. Youth provided valuable insight that they would be unlikely to click on a social media ad taking them to the intervention’s external website due to concern about false advertising. A systematic scoping review of interventions for adolescents with chronic health conditions [13], emphasized the importance of leveraging social media in future interventions rather than requiring an app download [14]. Interventions involving social media content are possible: adaptation of the #chatsafe guidelines for social media involved co-design with youth to create a resonant social media campaign about communicating safely about suicide on social media [15]. Youth involved in the co-design process also indicated a need for linked resources [15].

A limitation of this work is that we received feedback from only females and youth who reside in a small geographic area. Social desirability bias is likely due to the nature of the group setting. Minimizing social media harms has the potential to significantly influence a person’s life. An online self-guided SSI on this topic could promote equity by supplementing the heterogeneous information youth receive.

## Supplementary material

10.2196/73780 Multimedia Appendix 1 Focus group questions.

## Abbreviations

CIAS: computerized intervention authoring system
MI: motivational interviewing
SM SSI: social media single session intervention
SSI: single session interventions
