# Supplementary material for: A Social Media Single Session Intervention Designed to Promote Healthier Social Media Use: Youth Focus Group Feedback
Source: JMIR Form Res. 2026 Mar 2;10:e73780. doi: 10.2196/73780 (PMC12954684; doi:10.2196/73780)
Supplement: Multimedia Appendix 1 [file formative-v10-e73780-s001.docx]

Multimedia Appendix 1: Focus group questions

Pre SSI Questions:

1. **Have you learned about using social media in safe ways? If so, where (in school, in groups, with a medical provider, online etc.)?**
2. Are there things that you have done to try to change your social media use?
3. Do you talk with your parent or caregiver about your social media use?

Post SSI Questions:

**Questions adapted from the Program Feedback Scale**:

1. Did you enjoy this single session intervention? Why or why not?
2. What did you like about the single session intervention?
3. What would you change/improve about the single session intervention?
4. Did you understand this single session intervention? Why or why not?
5. Was doing the single session intervention easy to do? Why or why not?
6. Would you be able to focus during the single session intervention? Why or why not?
7. Do you think that the single session intervention would be helpful to other people your age? Why or why not?
8. Would you recommend this single session intervention to a friend? Why or why not?
9. Do you agree with the message of the single session intervention? Why or why not?

**Content questions:**

1. What are your initial reactions to the content of this single session intervention?
2. Did the stories seem real and relatable? Why or why not?
3. Was the number of stories about right or would you prefer to have more or less?
4. What do you think about the phrasing/wording throughout? Were there any instances where you thought words or phrases should be changed (either because they were wrong or not the words/phrases youth use today etc)
5. What is your opinion about the images and videos used in the single session intervention?
6. Is there anything about "being a teenager/young adult in the year 2024" that this activity may not fully consider or is not relevant to?
7. Is there any way we could make this activity more relevant to youth?
8. Did you learn something new while going through this single session intervention?

**Practical questions:**

1. Do you like the overall look/aesthetic of the single session intervention?
2. Was the single session intervention too long, too short, or just about the right length?
3. What feedback do you have about the logo?

**Implementation questions:**

1. How should this single session intervention be implemented? (Linked to a social media post, or stand-alone website, sent as a link via e-mail from school etc.)
2. What do you think will be the overall impact of doing this single session intervention for a person? How could it be more impactful?

**Other questions:**

1. What would you would like to get out of an intervention designed to help people with their social media use?
2. Are there other topics that would be more helpful that we didn’t include?
3. Would you do this single session intervention again on your own time? Why or why not?
4. What are your overall reactions to this single session intervention?
